# Cutaneous angiosarcoma treated with taxane‐based chemoradiotherapy: A multicenter study of 90 Japanese cases

**DOI:** 10.1002/ski2.180

**Published:** 2022-11-08

**Authors:** Taku Fujimura, Sadanori Furudate, Takeo Maekawa, Hiroshi Kato, Takamichi Ito, Shigeto Matsushita, Koji Yoshino, Akira Hashimoto, Yusuke Muto, Kentaro Ohuchi, Ryo Amagai, Yumi Kambayashi, Yasuhiro Fujisawa

**Affiliations:** ^1^ Department of Dermatology Tohoku University Graduate School of Medicine Sendai Japan; ^2^ Department of Dermatology Jichi Medical School Shimono Japan; ^3^ Department of Geriatric and Environmental Dermatology Nagoya City University Graduate School of Medical Sciences Nagoya Japan; ^4^ Department of Dermatology Graduate School of Medical Sciences Kyushu University Fukuoka Japan; ^5^ Department of Dermato‐Oncology/Dermatology National Hospital Organization Kagoshima Medical Center Kagoshima Japan; ^6^ Department of Dermato‐Oncology/Dermatology Cancer Institute Hospital of Japanese Foundation for Cancer Research Tokyo Japan; ^7^ Department of Dermatology University of Tsukuba Tsukuba Japan; ^8^ Department of Dermatology University of Ehime Matsuyama Japan

## Abstract

Cutaneous angiosarcoma (CAS) is rare and most previous studies of CAS have been small case series, and randomized, phase II studies of CAS are limited. Since treatment options for CAS are controversial, and because only paclitaxel should be recommended based on high‐level evidence, it is important to evaluate the efficacy of another taxane‐derived agents, docetaxel, in real‐world practice. The efficacy and safety profiles of chemoradiotherapy using taxane‐based agents, docetaxel and paclitaxel, were retrospectively examined in the maintenance setting in 90 Japanese CAS patients, including 35 docetaxel‐treated cases and 55 paclitaxel‐treated cases. Overall survival and dose duration time of the patient group treated with docetaxel was equivalent to that with paclitaxel, even in the cohorts with metastasis. Adverse events due to docetaxel and paclitaxel were observed in 77.1% and 69.1% of cases, respectively. The incidence ratio of total severe adverse events tended to be higher in the docetaxel‐treated group (40.0%) than in the paclitaxel‐treated group (23.6%). Peripheral neuropathy occurred only in the paclitaxel‐treated group, whereas high‐grade interstitial pneumonia developed only in the docetaxel‐treated group. In addition, we also evaluate 19 patients selected other taxanes, 17 patients selected eribulin methylate, 11 patients pazopanib, and 2 patients selected nivolumab as second‐line chemotherapy. The efficacy of a monthly docetaxel regimen is equivalent to a three‐weekly paclitaxel regimen evaluated by Overall survival and DDT, even in the cohorts with metastasis, and it is a tolerable protocol for CAS as a maintenance therapy in the Japanese population.

## Graphical abstract

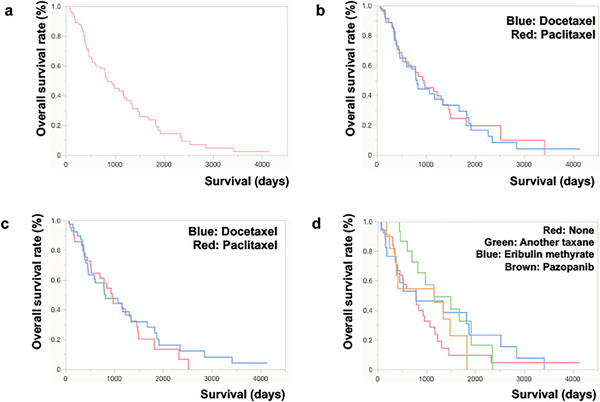

Although paclitaxel is given as a standard therapy for the treatment of cutaneous angiosarcoma (CAS), subsequent treatment for CAS is limited and still controversial. Several chemotherapy regimens were tentatively used for the treatment of CAS.In this report, the efficacy and safety profiles of monthly docetaxel regimen and a three‐weekly paclitaxel regimen in the maintenance setting after curative radiotherapy were evaluated retrospectively in 90 Japanese CAS patients. Our present study suggested that the efficacy of a monthly docetaxel regimen is equivalent to a three‐weekly paclitaxel regimen evaluated by OS and DDT, and is a tolerable protocol for CAS as a maintenance therapy.

**What is already known about this topic?**

- Although paclitaxel (PTX) is a standard therapy for the treatment of cutaneous angiosarcoma (CAS), subsequent treatment for CAS is limited and still controversial.
- Several chemotherapy regimens, such as docetaxel (DTX), eribulin and pazopanib, were tentatively used for the treatment of CAS as a second line chemotherapy.

**What does this study add?**

- The efficacy of a monthly docetaxel regimen is equivalent to a three‐weekly paclitaxel regimen evaluated by Overall survival (OS) and DDT, even in the cohorts with metastasis, and is a tolerable protocol for CAS as a maintenance therapy in the Japanese population.
- There is no significant different of OS between taxane‐resistant CAS patients who selected another taxanes, eribulin methylate or pazopanib as second‐line chemotherapy.

## 1 INTRODUCTION

Angiosarcoma, a rare malignancy of endothelial cells, develops mainly in the skin.^1^ CAS commonly occurs in the head and neck region in patients with no prior conditions. Soft‐tissue sarcoma accounts for only 1% of all malignant neoplasms^2^ and angiosarcoma comprises less than 2% of all soft‐tissue sarcomas.^3^, ^4^ Since CAS is rare, and since most previous studies of CAS have been small case series, randomized, phase II studies of CAS are limited.^5^, ^6^, ^7^ Therefore, no standardized treatment based on clinical trial evidence has been established.

Based on the previous clinical studies,^5^ chemoradiotherapy with paclitaxel are given as a standard therapy for the treatment of CAS.^8^ Other than taxane‐derived agents, vascular endothelial growth factor (VEGF) and VEGF receptor inhibitors or multi‐tyrosine kinase inhibitors (tyrosine kinase inhibitors (TKIs)) are permitted for the treatment of CAS.^8^, ^9^, ^10^ Several phase II trials of antiangiogenic TKIs for CAS have been reported, but the results were unsatisfactory.^10^ In addition to TKIs, recent reports suggested the effectiveness of immune checkpoint inhibitors for the treatment of CAS, especially with increased tumour mutation burden.^11^, ^12^ Eribulin mesylate is another option for the treatment of CAS in second‐line or beyond.^13^ Collectively, these reports suggest that the treatment options for CAS are controversial, and only taxane‐derived agents should be recommended based on high‐level evidence. Therefore, evaluations of the efficacy of taxane‐derived agents in real‐world practice are important. In this report, the efficacy and safety profiles of monthly docetaxel regimen and a three‐weekly paclitaxel regimen in the maintenance setting after curative radiotherapy were evaluated retrospectively in 90 Japanese CAS patients.

## 2 PATIENTS AND METHODS

### 2.1 Patients

A database generated by the dermatology departments at Tohoku University Graduate School of Medicine, Jichi Medical School, Nagoya City University Graduate School of Medical Sciences, Kyushu University, University of Tsukuba, the National Hospital Organisation Kagoshima Medical Centre, and the Cancer Institute Hospital of the Japanese Foundation for Cancer Research was retrospectively reviewed, and 90 patients with CAS who had been treated with taxane‐based chemotherapy in combination with radiotherapy between January 2010 and March 2022 were identified (Table 1). This study was approved by the institutional review board of Tohoku University Graduate School of Medicine (2022‐1‐096).

**TABLE 1:** Characteristics of patients with cutaneous angiosarcoma (CAS)

Median age, year	74.0 (34–93)
Sex	
Male	58
Female	32
Location	
Scalp	77
Face	2
Extremities	9
Trunk	2
Tumour stage	
Limited to the skin	84
Metastasis	6
First line chemotherapy	
Paclitaxel	55
Docetaxel	35

### 2.2 Treatment schedule and response assessment

Patients were intravenously administered taxane‐based chemoradiotherapy, docetaxel or paclitaxel, as a first‐line maintenance therapy in combination with radical radiotherapy (90 cases). Docetaxel was administered at 40–60 mg/m2 for a four‐week cycle. Paclitaxel was administered at 50–80 mg/m2 on days 1, 8, and 15 of a 28‐day cycle. Overall survival of all patients was evaluated as the primary endpoint. Dosing duration time (DDT), profiles of second‐line chemotherapy, and safety profiles were evaluated as secondary endpoints.

### 2.3 Safety assessment

Safety assessments involved the collection of data for severe adverse events (SAEs), results of clinical laboratory tests and physical examinations, and vital signs. Severity grade and categorisation was determined by Common Terminology Criteria for Adverse Events version 4.0—Japan Clinical Oncology Group.

### 2.4 Statistical analysis

Overall survival was defined as the time from the initial treatment date to the date of death. Overall survival was estimated using Kaplan‐Meier curves, and the log‐rank test was used to compare differences in OS. Wald Chi‐Square tests were used for multivariate analyses for OS and DDT (paclitaxel vs. docetaxel, tumour size, intensity modulated radiation therapy vs. stereotactic radiotherapy, dose of radiotherapy, metastasis, age, sex, and AEs). Each median was used for the cut off of tumour size, dose of radiotherapy and age. The 95% confidence interval (95% CI) for response was based on the binominal distribution. All statistical analyses were performed using JMP version 16.1 software (SAS Institute). The level of significance was set at *p* < 0.05.

## 3 RESULTS

### 3.1 Demographic data

Patient demographic data are shown in Table 1. The patients were 58 men and 32 women with a mean age of 73.5 years. The locations of CAS were the scalp (73 cases), face (5 cases), extremities (10 cases), and trunk (2 cases). Of the 90 patients, 6 had organ metastases at the initial visit. Of the 90 patients, 55 were given paclitaxel, and 35 were given docetaxel as first‐line chemoradiotherapy, and 19 patients selected surgical resection as a combination therapy with taxane‐based chemoradiotherapy (Table 1).

### 3.2 Efficacy

**FIGURE 1:**
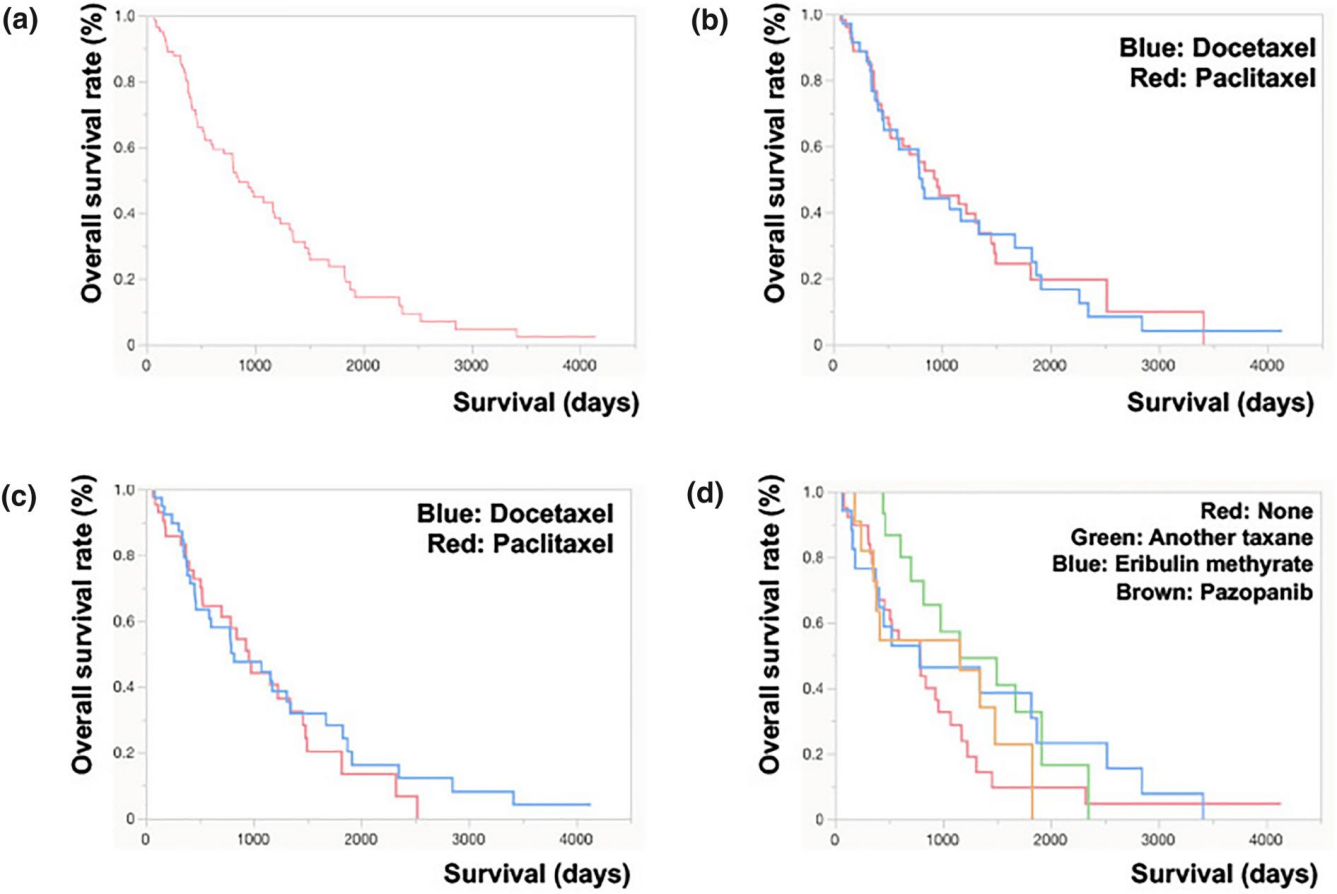
Overall survival (OS) of patients with cutaneous angiosarcoma (CAS). OS curves of 90 cases of CAS (a), with docetaxel and with paclitaxel in the entire cohort (b), and the cohort without metastasis (c). OS for each second‐line regimen (d)

Median survival time was 649 (61–4132) days, and 22 patients were still alive (Figure 1a). There was no significant difference between the patient group treated with docetaxel (792 days: *n* = 35) and the patient group with paclitaxel (624 days: *n* = 55) (*p* = 0.8854) (Figure 1b). For patients without metastasis at the initial visit, there was no significant difference between the patient group treated with docetaxel (793 days: *n* = 35) and the patient group with paclitaxel (565 days: *n* = 55) (*p* = 0.5855) (Figure 1c). A multivariate Wald Chip‐Square tests was used for the analysis of potential prognostic factors affecting OS of CAS, suggesting that selection of first line taxane, tumour size, protocol of radiotherapy, dose of radiotherapy, metastasis, age, sex, and AEs did not affect OS of CAS (Table 2). The median DDT of all taxane‐based chemotherapy cases (*n* = 90) was 274 (21–2906) days. There was no significant difference in DDT between the patient group treated with docetaxel (251 days: *n* = 35) and the patient group treated with paclitaxel (308 days: *n* = 55). A multivariate Wald Chi‐Square tests was used for the analysis of potential prognostic factors affecting DDT of CAS, suggesting was significantly correlate with the DDT of CAS (Table 2).

**TABLE 2:** A multivariate Wald Chi‐Square tests of potential prognostic factors affecting Overall survival (OS) (a) and DDT (b) of CAS

a		
	Wald Chi‐square	*p‐value*
PTX/DTX	1.548	0.214
Tumour size	0.019	0.89
IMRT/SRT	2.043	0.153
Dose of radiotherapy	0.006	0.938
Metastaiss	2.217	0.136
Age	3.167	0.075
Sex	0.557	0.455
AEs	2.067	0.151

b		
	Wald Chi‐square	*p‐value*
PTX/DTX	0.016	0.901
Tumour size	0.038	0.845
IMRT/SRT	1.193	0.275
Dose of radiotherapy	0.017	0.897
Metastaiss	0.045	0.832
Age	4.228	0.040
Sex	0.038	0.845
AEs	0.063	0.802

Abbreviations: DTX, docetaxel; IMRT, intensity modulated radiation therapy; PTX, paclitaxel; SRT, stereotactic radiotherapy.

### 3.3 Profiles of second‐line chemotherapy

Of the 90 patients, 49 underwent second‐line chemotherapy (Table 3). Nineteen patients selected other taxanes, 17 patients selected eribulin methylate, 11 patients pazopanib, and 2 patients selected nivolumab as second‐line chemotherapy. There was no significant difference in OS between the treated groups (*p* = 0.4047) (Figure 1d). The DDT and OS in each patient was described at supplemental figure.

**TABLE 3:** Profiles of second‐line chemotherapy for cutaneous angiosarcoma (CAS)

Second line therapy	All	PTX	DTX
Another taxanes	19	8	11
Eribulin methylate	17	13	4
Pazopanib	11	5	6
Nivolumab	2	2	0
None	41	27	14

Abbreviations: DTX, docetaxel; PTX, paclitaxel.

### 3.4 Safety profile

Safety profiles for each cohort are shown in Table 4. The incidence of all AEs was 72.2% (95% CI: 62.1%–80.5%). The frequency of all AEs in the paclitaxel‐treated group was 69.1% (95% CI, 55.9%–79.8%), and that of the docetaxel‐treated group was 77.1% (95% CI: 60.8%–88.2%). The incidence of all SAEs for all patients was 23.6% (95% CI: 14.2%–36.5%), including 14 cases of leucopenia, 8 cases of neutropenia, and three cases of interstitial pneumonia. The frequency of SAEs in the paclitaxel‐treated group was 23.6% (95% CI, 14.2%–36.5%), and that of the docetaxel‐treated group was 40.0% (95% CI: 25.5%–56.5%).

**TABLE 4:** Safety profile

		PTX	DTX
G1/G2 adverse events			
Peripheral neuropathy	12	12	0
Neutropenia	9	5	4
Interstitial pneumonia	7	5	2
Leucopenia	5	2	3
Hepatitis	4	1	3
Anorexia	3	2	1
Diarrhoea	2	0	2
Anaemia	2	0	2
Stomatitis	1	0	1
Pancytopenia	1	1	0
Thrombocytopaenia	1	1	0
Melaise	1	1	0
Nail dropout	1	1	0
Constipation	1	1	0
Pleural effusion	1	0	1
Skin eruption	1	0	1
G3/G4 severe adverse events			
Leucopenia	14	2	12
Neutropenia	8	6	2
Interstitial pneumonia	3	0	3
Pancytopenia	2	2	0
Pleural effusion	1	1	0
Artioventicular block	1	1	0
Skin eruption	1	1	0
Thrombocytopaenia	1	0	1
Infection	1	1	0
Hepatitis	1	0	1
Hyperkalemia	1	0	1

Abbreviations: DTX, docetaxel; PTX, paclitaxel.

## 4 DISCUSSION

Cutaneous angiosarcoma is a highly aggressive type of vascular tumour, histologically characterised by detachment of endothelial cell‐derived tumour cells.^14^, ^15^ Since there is no standardized treatment algorithm for each stage, staging of CAS has little clinical benefit in treatment decision‐making.^14^ Therefore, it is extremely important for oncologists to estimate OS of CAS treated with standard therapy in real‐world practice. Recent clinical studies have suggested the importance of taxane‐derived agents such as paclitaxel and docetaxel as first‐line chemotherapy for the treatment of CAS.^5^, ^8^ Of them, a phase II study of weekly paclitaxel for the treatment of unresectable CAS suggested the clinical benefit of paclitaxel in CAS.^5^ In addition, chemoradiotherapy with taxanes achieved a high response rate, with significant improvement of OS compared with surgery or radiotherapy.^8^ From the above findings, taxane‐based chemoradiotherapy is administered as a first‐line standard therapy for the treatment of CAS in Japan. Although the clinical benefits of paclitaxel and docetaxel for the treatment of CAS are widely recognized, no report has compared the efficacy of paclitaxel and docetaxel in combination with radiotherapy as a first‐line therapy for CAS.

In this report, the efficacy and safety profiles of docetaxel and paclitaxel in the maintenance setting were retrospectively analysed in 90 Japanese CAS patients, including 35 docetaxel‐treated cases and 55 paclitaxel‐treated cases. The efficacy of monthly docetaxel regimen is equivalent to three‐weekly paclitaxel evaluated by OS and DDT, even in the cohorts with metastasis. These results suggested that docetaxel could also be a first‐line chemoradiotherapy for the treatment of CAS.

Paclitaxel and docetaxel not only induce tumour cytotoxicity, but also modulate the tumour microenvironment to induce anti‐tumour immune responses and anti‐angiogenetic reactions in various cancer species.^16^, ^17^, ^18^, ^19^ Low‐dose docetaxel augments the anti‐tumour immune response of endothelial cell vaccine by the induction of endothelial‐specific CD8+ cytotoxic T cells in an ET1 mouse breast cancer model in vivo, suggesting that docetaxel could not only enhance the anti‐tumour immune response, but also inhibit angiogenesis in breast cancer.^16^ In addition, docetaxel suppresses the recruitment of regulatory T cells (Tregs) in the tumour microenvironment,^16^ which might be caused by its immunomodulatory effects on tumour‐associated macrophages.^17^ Indeed, docetaxel decreased the production of Treg‐related chemokines such as CCL22 and CCL18 from monocyte‐derived M2 macrophages in vitro.^17^ These reports suggested the immunomodulatory and anti‐angiogenesis effects of docetaxel. Paclitaxel also increased CD8+ T cells through toll‐like receptor4‐dependent pathways in an ovarian cancer model.^18^ Importantly, TLR4 stimulation activated perivascular macrophages to induce endothelial cell‐mediated production of a pro‐metastatic vascular niche during breast cancer colonisation in the lung,^19^ suggesting that paclitaxel might be useful for the induction of anti‐tumour immune responses against cancers, but it also induces angiogenesis to promote cancer development. Indeed, paclitaxel interferes with endothelial tip cells promoted by VEGF signalling to suppress their sprouting behaviour, but allow their lumen formation.^20^ Notably, a previous clinical trial suggested that the overall response rate of bevacizumab, an anti‐VEGF Ab, was 13%, indicating that VEGF signal blockade is likely insufficient for the treatment of metastatic or locally advanced CAS.^21^ In consideration of these findings from other studies, the different additional effects on the tumour microenvironment between paclitaxel and docetaxel might have impacted OS in each group of the present study.

Adverse events due to docetaxel and paclitaxel were observed in 77.1% and 69.1%, respectively. Peripheral neuropathy occurred only in the paclitaxel‐treated group. In contrast, the incidence ratio of total SAEs tended to be higher in the docetaxel‐treated group (40.0%) than in the paclitaxel‐treated group (23.6%). Notably, high‐grade interstitial pneumonia developed only in the docetaxel‐treated group. This tendency might be caused by the different chemotherapy protocol; docetaxel is given monthly, whereas paclitaxel is given as a three‐weekly course. Overall, both paclitaxel and docetaxel are a tolerable protocol for CAS as a maintenance therapy in the Japanese population. Since this study is retrospective, observational study, further prospective, randomized clinical trial is needed to confirm the safety and efficacy of docetaxel for CAS in future.

## CONFLICTS OF INTEREST

The authors declare that there is no conflict of interest that could be perceived as prejudicing the impartiality of the research reported.

## AUTHOR CONTRIBUTIONS

**Taku Fujimura**: Data curation (Equal); Formal analysis (Equal); Resources (Equal); Supervision (Equal); Writing – original draft (Lead); Writing – review & editing (Lead). **Sadanori Furudate**: Data curation (Equal); Formal analysis (Equal); Resources (Equal). **Takeo Maekawa**: Data curation (Equal); Formal analysis (Equal); Resources (Equal). **Hiroshi Kato**: Data curation (Equal); Formal analysis (Equal); Resources (Equal). **Takamichi Ito**: Data curation (Equal); Formal analysis (Equal); Resources (Equal). **Shigeto Matsushita**: Data curation (Equal); Formal analysis (Equal); Resources (Equal). **Koji Yoshino**: Data curation (Equal); Formal analysis (Equal); Resources (Equal). **Akira Hashimoto**: Data curation (Equal); Formal analysis (Equal); Resources (Equal). **Yusuke Muto**: Data curation (Equal); Formal analysis (Equal); Resources (Equal). **Kentaro Ohuchi**: Formal analysis (Equal); Funding acquisition (Equal); Resources (Equal). **Ryo Amagai**: Data curation (Equal); Formal analysis (Equal); Resources (Equal). **Yumi Kambayashi**: Data curation (Equal); Formal analysis (Equal); Resources (Equal). **Yasuhiro Fujisawa**: Data curation (Equal); Formal analysis (Equal); Resources (Equal); Supervision (Equal).

## ETHICS STATEMENT

Written, informed consent was obtained from the patient for publication of this case report and any accompanying images. The protocol for this human study was approved by the ethics committee of Tohoku University Graduate School of Medicine, Sendai, Japan (permit no. 2022‐1‐096).

## Supporting information

Supporting Information S1

## Data Availability

All data generated or analysed during this study are included in this article. Further enquiries can be directed to the corresponding author.
