# Supplementary material for: Cutaneous angiosarcoma treated with taxane‐based chemoradiotherapy: A multicenter study of 90 Japanese cases
Source: Skin Health Dis. 2022 Nov 8;3(1):e180. doi: 10.1002/ski2.180 (PMC9892413; doi:10.1002/ski2.180)

Supplemental table: Characteristics of patients with CAS

| <!--Col Count:9--> | Age | Sex | Location | Tumor size (mm) | Radioterapy | Total dose (Gy) | Metastasis | first line |
| --- | --- | --- | --- | --- | --- | --- | --- | --- |
| Case 1 | 79 | M | Scalp | 55 | SRT | 30 | - | PTX |
| Case 2 | 70 | F | Scalp | 60 | SRT | 70 | - | DTX |
| Case 3 | 70 | F | Scalp | 65 | SRT | 60 | - | DTX |
| Case 4 | 68 | M | Scalp | 110 | SRT | 70 | - | DTX |
| Case 5 | 83 | M | Cheek | 65 | SRT | 70 | - | DTX |
| Case 6 | 67 | M | Scalp | 30 | SRT | 70 | - | PTX |
| Case 7 | 93 | M | Cheek | 90 | SRT | 70 | - | PTX |
| Case 8 | 70 | M | Scalp | 50 | SRT | 70 | - | PTX |
| Case 9 | 83 | F | Scalp | 15 | SRT | 70 | - | PTX |
| Case 10 | 72 | M | Scalp | 30 | SRT | 70 | - | PTX |
| Case 11 | 69 | M | Scalp | 20 | SRT | 70 | - | DTX |
| Case 12 | 78 | M | Scalp | 25 | SRT | 70 | - | DTX |
| Case 13 | 84 | M | Scalp | 80 | SRT | 70 | - | DTX |
| Case 14 | 87 | M | Scalp | 33 | SRT | 70 | - | PTX |
| Case 15 | 67 | M | Scalp | 60 | SRT | 70 | - | DTX |
| Case 16 | 72 | M | Scalp | 80 | SRT | 70 | - | PTX |
| Case 17 | 71 | F | Scalp | 130 | SRT | 70 | - | DTX |
| Case 18 | 86 | M | Scalp | 47 | SRT | 72.5 | - | DTX |
| Case 19 | 77 | M | Scalp | 120 | SRT | 70 | - | DTX |
| Case 20 | 74 | M | Scalp | 76 | SRT | 65 | - | DTX |
| Case 21 | 57 | F | Scalp | 46 | SRT | 64.8 | - | PTX |
| Case 22 | 76 | M | Scalp | 74 | SRT | 70 | - | PTX |
| Case 23 | 68 | F | Scalp | 52 | SRT | 70 | - | PTX |
| Case 24 | 77 | F | Scalp | 96 | SRT | 70 | Lung | PTX |
| Case 25 | 67 | M | Scalp | 38 | SRT | 70 | - | PTX |
| Case 26 | 72 | M | Scalp | 60 | SRT | 65 | - | PTX |
| Case 27 | 79 | F | Scalp | 44 | SRT | 60 | - | PTX |
| Case 28 | 65 | F | Lower extremities | 190 | SRT | 72 | - | PTX |
| Case 29 | 81 | F | Scalp | 66 | SRT | 52.5 | - | PTX |
| Case 30 | 74 | F | Scalp | 130 | SRT | 60 | - | PTX |
| Case 31 | 80 | M | Scalp | 100 | SRT | 70 | - | PTX |
| Case 32 | 65 | M | Scalp | 78 | SRT | 60 | - | PTX |
| Case 33 | 69 | F | Scalp | 122 | SRT | 60 | - | PTX |
| Case 34 | 87 | M | Scalp | 47 | SRT | 60 | - | PTX |
| Case 35 | 81 | M | Scalp | 76 | IMRT | 66 | - | PTX |
| Case 36 | 70 | F | Shoulder | 100 | SRT | 66 | - | PTX |
| Case 37 | 72 | M | Scalp | 186 | IMRT | 60 | - | PTX |
| Case 38 | 83 | F | Scalp | 138 | IMRT | 66 | - | PTX |
| Case 39 | 74 | M | Scalp | 30 | SRT | 70 | - | PTX |
| Case 40 | 74 | M | Scalp | 100 | SRT | 70 | - | PTX |
| Case 41 | 77 | M | Scalp | 130 | SRT | 62 | - | PTX |
| Case 42 | 65 | F | Lower extremities | 60 | SRT | 70 | - | PTX |
| Case 43 | 77 | M | Scalp | 150 | SRT | 70 | - | PTX |
| Case 44 | 59 | M | Scalp | 20 | SRT | 40 | - | PTX |
| Case 45 | 76 | F | Scalp | 25 | SRT | 70 | - | PTX |
| Case 46 | 76 | F | Scalp | 40 | SRT | 70 | - | PTX |
| Case 47 | 82 | M | Scalp | 25 | SRT | 70 | - | PTX |
| Case 48 | 47 | M | Scalp | 40 | IMRT | 70 | - | PTX |
| Case 49 | 80 | M | Scalp | 110 | SRT | 70 | - | PTX |
| Case 50 | 80 | M | Scalp | 40 | IMRT | 70 | Liver, Spleen | DTX |
| Case 51 | 61 | M | Scalp | 40 | IMRT | 70 | - | DTX |
| Case 52 | 70 | M | Scalp | 50 | IMRT | 70 | - | DTX |
| Case 53 | 75 | M | Scalp | 18 | IMRT | 70 | - | DTX |
| Case 54 | 64 | F | Scalp | 40 | IMRT | 70 | - | DTX |
| Case 55 | 64 | M | Scalp | 30 | IMRT | 70 | - | DTX |
| Case 56 | 77 | M | Scalp | 70 | IMRT | 70 | - | DTX |
| Case 57 | 83 | F | Scalp | 50 | IMRT | 70 | - | DTX |
| Case 58 | 72 | M | Scalp | 35 | IMRT | 70 | - | DTX |
| Case 59 | 70 | M | Scalp | 50 | IMRT | 70 | - | DTX |
| Case 60 | 76 | M | Scalp | 13 | IMRT | 64 | - | DTX |
| Case 61 | 73 | M | Scalp | 90 | IMRT | 70 | - | PTX |
| Case 62 | 77 | M | Scalp | 50 | IMRT | 70 | - | DTX |
| Case 63 | 74 | F | Scalp | 60 | IMRT | 70 | - | DTX |
| Case 64 | 76 | F | Lower extremities | 50 | IMRT | 60 | - | DTX |
| Case 65 | 59 | F | Lower extremities | 50 | IMRT | 60 | Inguinal lymph nodes | DTX |
| Case 66 | 78 | F | Scalp | 50 | IMRT | 70 | - | PTX |
| Case 67 | 34 | M | Lower extremities | 57 | IMRT | 70 | - | DTX |
| Case 68 | 72 | M | Scalp | 50 | IMRT | 70 | Cervical lymph nodes | PTX |
| Case 69 | 84 | M | Scalp | 50 | IMRT | 27 | Liver | PTX |
| Case 70 | 68 | M | Scalp | 90 | IMRT | 70 | - | DTX |
| Case 71 | 76 | F | Scalp | 90 | SRT | 66 | - | DTX |
| Case 72 | 95 | F | Scalp | 60 | SRT | 60 | - | DTX |
| Case 73 | 69 | M | Scalp | 50 | SRT | 69 | - | DTX |
| Case 74 | 72 | M | Scalp | 20 | SRT | 66 | - | DTX |
| Case 75 | 74 | F | Scalp | 11 | SRT | 66 | - | PTX |
| Case 76 | 81 | M | Scalp | 20 | SRT | 70 | - | PTX |
| Case 77 | 70 | M | Scalp | 30 | IMRT | 66 | - | PTX |
| Case 78 | 62 | F | Scalp | 80 | IMRT | 70 | - | PTX |
| Case 79 | 75 | M | Scalp | 20 | IMRT | 66 | - | PTX |
| Case 80 | 64 | F | Scalp | 100 | IMRT | 70 | Lung | PTX |
| Case 81 | 78 | M | Scalp | 150 | IMRT | 60 | - | PTX |
| Case 82 | 73 | M | Scalp | 35 | IMRT | 66 | - | DTX |
| Case 83 | 74 | M | Scalp | 30 | IMRT | 60 | - | DTX |
| Case 84 | 72 | F | Scalp | 150 | IMRT | 70 | - | PTX |
| Case 85 | 73 | M | Scalp | 85 | IMRT | 70 | - | PTX |
| Case 86 | 72 | M | Scalp | 180 | IMRT | 70 | - | PTX |
| Case 87 | 80 | M | Scalp | 40 | IMRT | 70 | - | PTX |
| Case 88 | 60 | M | Lower extremities | 200 | IMRT | 66 | - | PTX |
| Case 89 | 78 | F | Upper extremities | 400 | IMRT | 68 | - | PTX |
| Case 90 | 75 | F | Upper extremities | 50 | IMRT | 60 | - | PTX |

Supplemental figure

DDT of second line therapy in each case. Arrow: treatment is still ongoing.


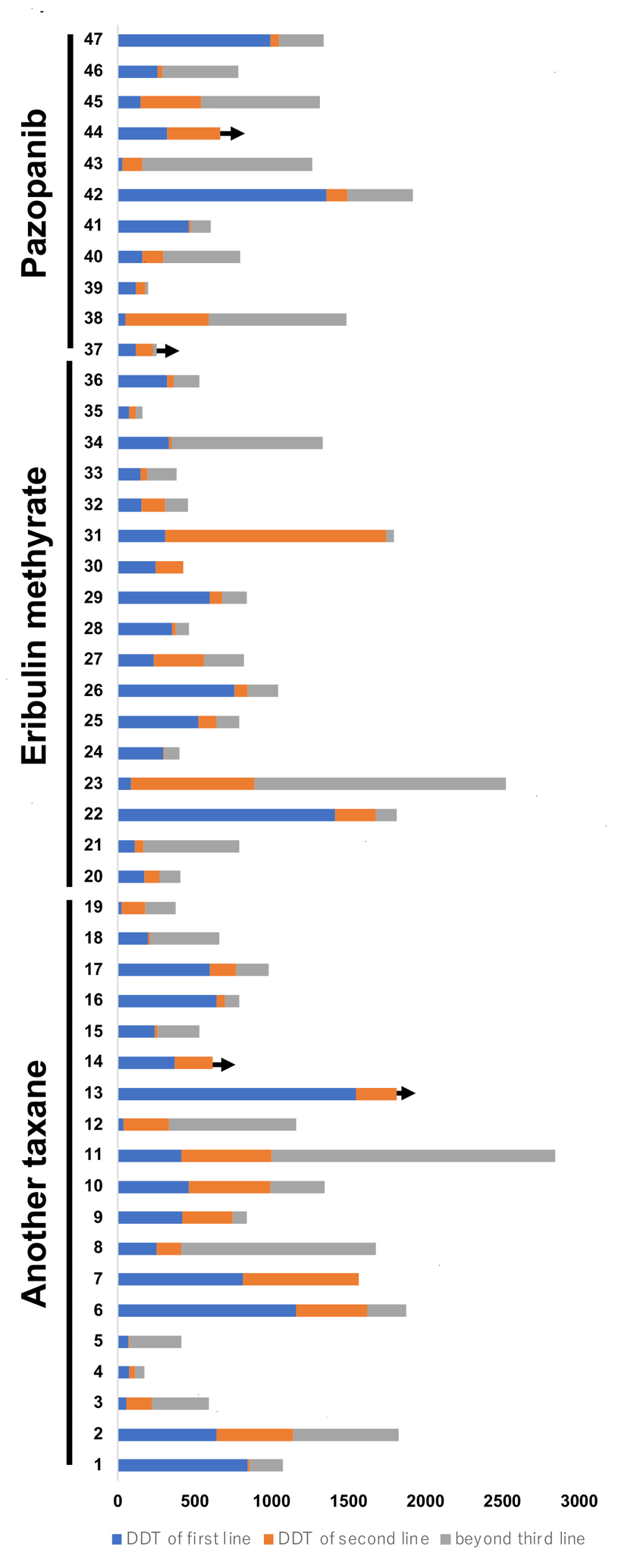

Supplement: Supplementary file 1 — Supporting Information S1 [file SKI2-3-e180-s001.docx]
